# A Vertex Model of *Drosophila* Ventral Furrow Formation

**DOI:** 10.1371/journal.pone.0075051

**Published:** 2013-09-16

**Authors:** Philipp Spahn, Rolf Reuter

**Affiliations:** Interfakultäres Institut für Zellbiologie, Abteilung Genetik der Tiere, Fachbereich Biologie, Universität Tübingen, Germany; University of Cambridge, United Kingdom

## Abstract

Ventral furrow formation in *Drosophila* is an outstanding model system to study the mechanisms involved in large-scale tissue rearrangements. Ventral cells accumulate myosin at their apical sides and, while being tightly coupled to each other via apical adherens junctions, execute actomyosin contractions that lead to reduction of their apical cell surface. Thereby, a band of constricted cells along the ventral epithelium emerges which will form a tissue indentation along the ventral midline (the ventral furrow). Here we adopt a 2D vertex model to simulate ventral furrow formation in a surface view allowing easy comparison with confocal live-recordings. We show that in order to reproduce furrow morphology seen *in vivo*, a gradient of contractility must be assumed in the ventral epithelium which renders cells more contractile the closer they lie to the ventral midline. The model predicts previous experimental findings, such as the gain of eccentric morphology of constricting cells and an incremental fashion of apical cell area reduction. Analysis of the model suggests that this incremental area reduction is caused by the dynamical interplay of cell elasticity and stochastic contractility as well as by the opposing forces from contracting neighbour cells. We underpin results from the model through *in vivo* analysis of ventral furrow formation in wildtype and *twi* mutant embryos. Our results show that ventral furrow formation can be accomplished as a “tug-of-war” between stochastically contracting, mechanically coupled cells and may require less rigorous regulation than previously thought.

**Summary:**

For the developmental biologist it is a fascinating question how cells can coordinate major tissue movements during embryonic development. The so-called ventral furrow of the Drosophila embryo is a well-studied example of such a process when cells from a ventral band, spanning nearly the entire length of the embryo, undergo dramatic shape change by contracting their tips and then fold inwards into the interior of the embryo. Although numerous genes have been identified that are critical for ventral furrow formation, it is an open question how cells work together to elicit this tissue rearrangement. We use a computational model to mimic the physical properties of cells in the ventral epithelium and simulate the formation of the furrow. We find that the ventral furrow can form through stochastic self-organisation and that previous experimental observations can be readily explained in our model by considering forces that arise when cells execute contractions while being coupled to each other in a mechanically coherent epithelium. The model highlights the importance of a physical perspective when studying tissue morphogenesis and shows that only a minimal genetic regulation may be required to drive complex processes in embryonic development.

## Introduction

Gastrulation is the first major morphogenetic event during *Drosophila* embryogenesis and an outstanding model system to address the mechanisms by which cell shape changes evoke a large-scale tissue rearrangement. During a remarkably fast time-span of about 10 minutes ventral cells constrict their apices and form an indentation in the ventral epithelium (the ventral furrow) which subsequently invaginates into the interior of the embryo to commence the development of mesodermal structures (for a review see [1]). Apical constriction is facilitated as myosin is specifically relocalized to the apices in ventral cells [2]. This relocalization depends on RhoGEF2 [2], [3] which itself accumulates apically through the combined action of Folded gastrulation (Fog) and T48. The ventral expression of these factors in turn depends on Twist (Twi) [2], [4], [5]. The role of the other major mesodermal determinant Snail (Sna) remains still largely unclear. Apical actomyosin assembles into a meshwork spanning the inner apical cell membrane and contracts in discontinuous, stochastic pulses to reduce the apical cell surface [6]. The contraction force is translated into cell shape change by apical adherens junctions linking the actomyosin to the cell membranes [2], [6]–[9]. Although much progress has been made identifying the genetic players involved in apical constriction, it is not clear what essential regulatory inputs are required to make cells of the ventral epithelium undergo a joint constriction, namely the formation of a band of constricted cells.

Computational modelling is a premier method to address this issue since simulating a complex process *in silico* can clarify which mechanisms are critical to explain *in vivo* observations or whether postulates made from experimental data may be expendable. Several computational approaches have been undertaken to address the biophysical implications of *Drosophila* gastrulation, mostly by computer simulation of 2D-representations of the embryo in cross-section [10]–[15]. Even the feasibility to simulate furrow invagination in a 3D computer model has been successfully demonstrated [16]–[18]. These computational studies have greatly advanced the understanding of the combinatorial effect of physical forces arising both in the ventral and lateral epithelium to enable tissue invagination. In addition, they have made aware that the unravelling of an inherently biophysical process like the invagination of the ventral furrow cannot be fully achieved without utilizing biophysical and computational approaches.

**Figure 1:**
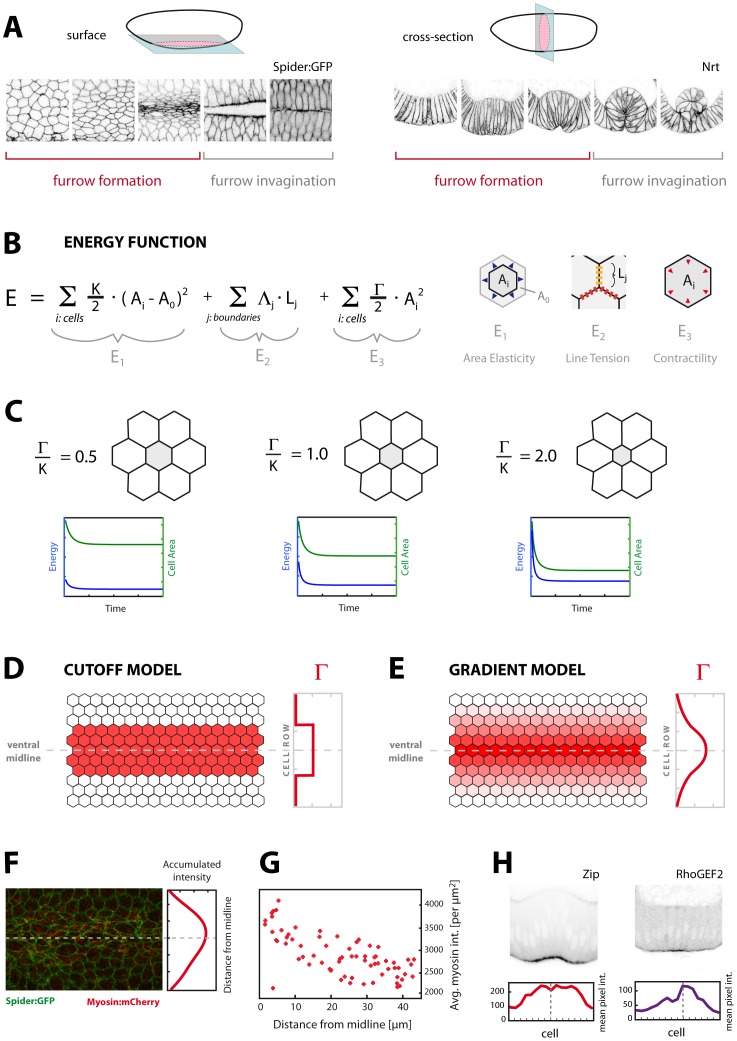
A surface-view computational model of ventral furrow formation. **A:** Surface sections (left) and cross-sections (right) of the ventral epithelium of the *Drosophila* embryo during ventral furrow formation and furrow invagination. Image series span approximately 20 minutes real-time (Nrt: Neurotactin). **B:** Energy contributions considered for the vertex model of the epithelium in surface view. E_1_ represents area elasticity with A_i_ being the surface area of cell *i* and A_0_ its preferred area. As no experimental data would suggest otherwise, the elasticity parameter K and preferred area A_0_ are assumed equal for all cells. E_2_ represents line tension arising along cell-cell boundaries. Transverse tensions (red in the cartoon) are assumed higher than vertical tensions (yellow in the cartoon) according to results shown in [21], [30]. E_3_ represents contractility (preferred area equals zero in this case). **C:** Reduced model with seven cells with different ratios of contractility and area elasticity in the central cell (grey). Contractility is set to zero in the surrounding cells. The amount of constriction achieved at force balance ( = minimized energy) depends on the ratio of contractility and area elasticity. **D:** Cutoff model of contractility: Cells in an antero-posterior strip on the ventral epithelium have equal non-zero contractility while cells lying outside are non-contractile. **E:** Gradient model of contractility: Contractility gradually decreases the further away a cell is located from the ventral midline. **F:** Still of a confocal live-recording with myosin labelled in red (green: Spider:GFP). Accumulated myosin intensity is measured by integrating pixel intensity for each pixel row in the image (dotted line: ventral midline). **G:** Scatter plot of myosin intensity (per µm^2^), averaged over the time of furrow formation, against cell distance from the ventral midline. The closer the cell lies to the midline, the higher is its average myosin intensity over time. **H:** Cross-sections of embryos fixed during ventral furrow formation and immunostained for Zipper (myosin, left) or RhoGEF2 (right). For each cell (identified in the parallel channel with membranes labelled by anti-Nrt, not shown) an apical ROI was drawn and mean pixel intensity was measured.

To keep the nomenclature consistent throughout this article, we would like to clearly differentiate between successive stages of *Drosophila* gastrulation (reviewed in [1]) and use the term “ventral furrow formation” only to describe the early stage of gastrulation, spanning from the completion of cellularization to the formation of a band of constricted cells along the ventral epithelium. In particular, we prefer to clearly separate “furrow formation” from “furrow invagination” that refers to the subsequent stage when the mesoderm folds into the interior of the embryo (Fig. 1A). While the biomechanics of furrow invagination have been thoroughly addressed as mentioned above, the early phase of gastrulation, i.e. the emergence of a ventral band of constricted cells, has not caught much attention yet in computational modelling. Martin and colleagues [6] proposed a ratchet model of apical constriction stating that cells undergo a few repeated cycles (3,2±1,2 on average) of contraction and stabilization to incrementally reduce their apical surface, but this model is not computational. Driquez and colleagues [19] present 1D and 2D computational models, largely based on data in [20] and [6] assuming a two-phase process where Sna–dependent random oscillations trigger a second, Fog–dependent phase to constrict the apical surfaces in a rigorously regulated fashion. A direct representation of the simulated apical surface view of the ventral epithelium is not provided, however.

In this project we aimed at building a computational model that would allow direct visual and quantitative comparison to confocal live-recordings taken in surface view. Critical events associated with furrow formation (actomyosin contraction, adherens junction assembly, apical constriction) take place at the apical side of ventral cells, so imaging of surface sections was frequently used as the method of choice to investigate the cell biological mechanisms of ventral furrow formation (e.g.: [6], [7], [9], [21], [22]). We applied a vertex model to describe the cell sheet of the *Drosophila* ventral epithelium and sought to elucidate what input would be required to make the epithelium form the ventral furrow. We find that the ventral furrow can be well reproduced if ventral cells exhibit a stochastic contractility and cell contractility follows a gradient rising from lateral towards the ventral midline. The model proves to have predictive capability since much-noticed phenomena, such as the emergence of eccentric cell morphology [21] and an incremental cell surface reduction [6] seem to implicitly follow from our model set-up. In particular, the incremental reduction of surface area appears to be a consequence of counteracting elastic and contractive forces, both within the cell itself as well as between adjacent cells. Considering our model analysis in combination with live-recordings of wildtype and *twi* mutant embryos, we discuss a parsimonious concept of furrow formation.

## Results

### A Vertex Model for Ventral Furrow Formation

The ventral epithelium of the *Drosophila* embryo in surface view is modelled as a sheet of hexagonal cells. We use a vertex model to describe the components of potential energy relevant to the cellular events during furrow formation (Fig. 1B). Similar approaches have been used previously to model cell sheets connected through adherens junctions in various developmental contexts [23]–[29]. In particular, the energy contributions considered are: area elasticity, line tension, and contractility (Fig. 1B). Area elasticity describes energy required to expand or to compress the cell’s surface area with respect to its preferred area (A_0_). Line tension refers to tension arising along cell-cell boundaries. In order to account for previous findings revealing asymmetrical tension across the tissue during ventral furrow formation [21], [30], we let “transverse” line tension (across the antero-posterior axis) be higher than “vertical” line tension (across the latero-ventral axis) (Fig. 1B). Finally, contractility accounts for the actomyosin contractions driving apical cell constriction. Other than in previous variants of this vertex model we let contractile energy depend on cell area rather than cell perimeter since actomyosin contractions during ventral furrow formation have been shown to occur across the apical cell surface and are not restricted to a circumferential actomyosin ring [6].

Starting from a relaxed condition, i.e. cells having their preferred area and preferred boundary lengths, contractility makes cells constrict their area until contractile and elastic forces balance each other and a local energy minimum is reached. The degree of constriction largely depends on the ratio of contractility and area elasticity (Fig. 1C). We prefer to strictly discern between the notions “contraction” and “constriction” as we use “contraction” to describe intrinsic actomyosin contractile activity and “constriction” to refer to the observable effect of this contractility on cell shape, i.e. apical area reduction. Due to the opposing elastic forces, enduring contractility does not necessarily imply enduring constriction as the cell will finally arrive in a state where force balance is reached (Fig. 1C). To explore how an entire cell sheet could undergo the large-scale morphological change observed during ventral furrow formation, we impose a contractility function upon the sheet that would be allowed to vary in space and time. We assume that cells have taken on their preferred area and preferred boundary lengths in the mostly regular arrangement after completion of cellularization, so by enabling contractility, cells will be forced to undergo constriction.

**Figure 2:**
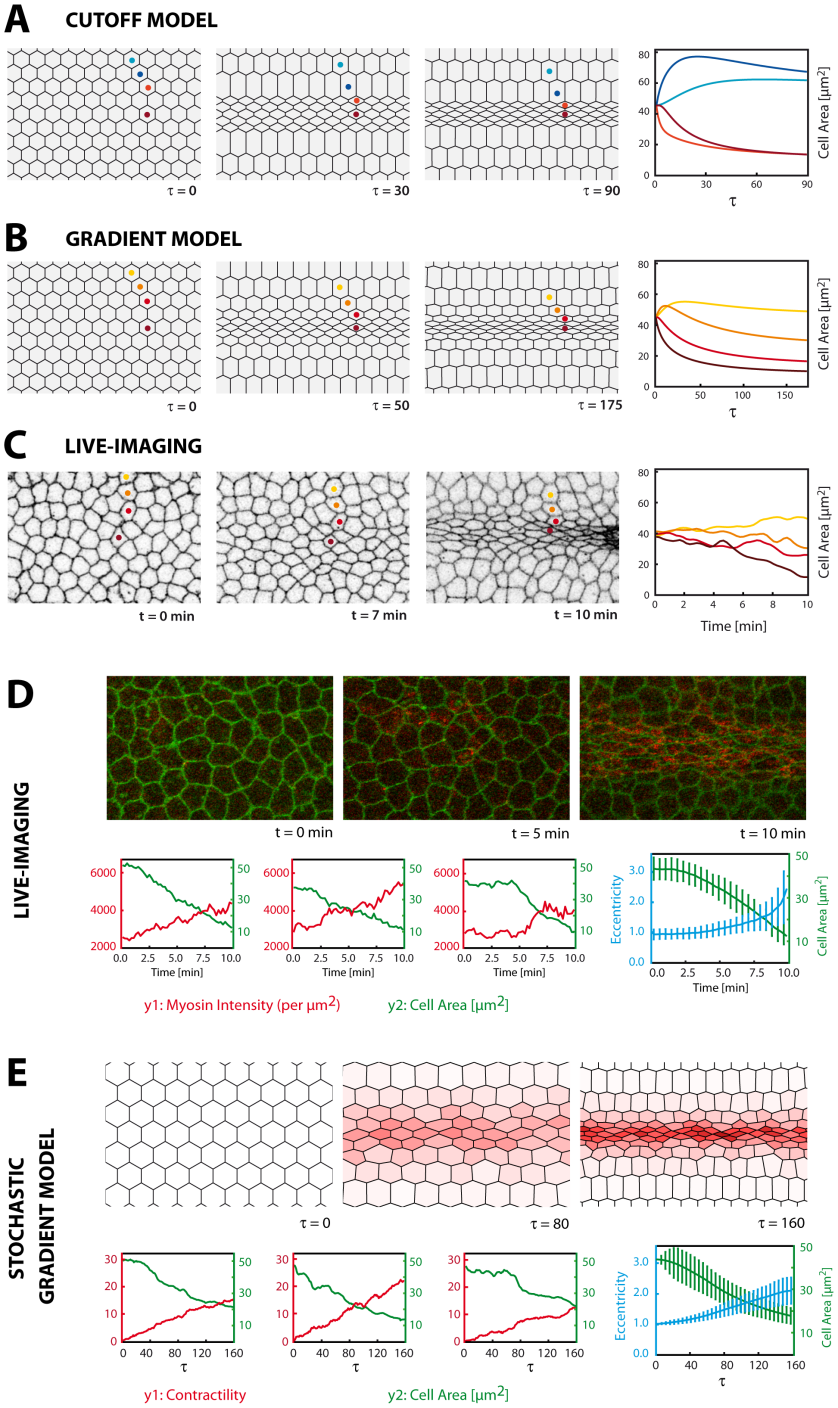
Reproduction of ventral furrow formation. **A–C:** Morphology of the ventral epithelium in the cutoff (A) or gradient (B) model with plots of apical area of indicated cells. In the gradient model constriction levels gradually decrease with distance from the ventral midline (B), comparable to live-imaging (C). In the cutoff model (A), constricted cells ( = contractile cells, red) directly abut unconstricted cells ( = non-contractile cells, blue). 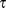 refers to time-steps in the model. Area designations in µm^2^ are obtained after normalization to live-imaging data (see Materials & Methods). **D:** Live-recording of furrow formation spanning 10 minutes real-time with labelled membranes (green: Spider:GFP) and myosin (red: sqh:mCherry). Myosin contraction occurs in a stochastic fashion and autonomously in each cell. Plots depict cell area and myosin intensity in three cells. Right plots depicts cell area and eccentricity (see Fig. 3A), averaged over all ventral cells (mean ± s.d.). **E:** Simulation of the gradient model with time-dependent, stochastic contractility (coded by colour) ( = stochastic gradient model). Plots depict cell area and contractility in three cells. Right plots depicts cell area and eccentricity, averaged over all ventral cells (mean ± s.d.).

In gastrulating embryos, constriction only occurs in ventral cells (defined here as all cells which become internalized during furrow invagination). However, among these cells different constriction levels are achieved since cells lying closer to the ventral midline yield higher constriction than those lying further lateral before they become invaginated [22], [31], see also Figs. 1A, 2C). This prompts the question whether all ventral cells have the same contractility or whether contractility varies among them. Assuming temporarily constant contractility in a first approach, we set up two model variants with regard to how contractility varies spatially within the sheet. It may be that only a narrow antero-posterior strip of ventral cells is contractile, while the remaining ventral cells are non-contractile (as are lateral cells). Alternatively, contractility may gradually decline the further away a cell is located from the ventral midline. Accordingly, in the first variant (named “Cutoff”) a sharp boundary between contractile and non-contractile cells is assumed: Central cells are contractile (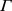
>0) while the remainder is non-contractile (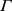 = 0) (Fig. 1D). In the alternative model variant (named “Gradient”), contractility gradually decreases from the ventral midline towards lateral cells (Fig. 1E). Experimental evidence supports this notion of a contractility gradient as intensity measurements of apical myosin reveal a gradual decrease from ventral to lateral during furrow formation (Fig. 1F–H), consistent with a gradual decrease of constriction levels achieved [22]. Interestingly, upstream regulators of myosin activation, such as RhoGEF2 (Fig. 1H), Fog [4], and Twi [32] also show a gradual expression in the ventral epithelium. These data would favour the hypothesis of a ventral-to-lateral contractility gradient, but, nonetheless, both model variants are implemented and tested for their performance.

### A Contractility Gradient, Combined with Stochastic Contraction Dynamics, Accurately Reproduces Ventral Furrow Formation

**Figure 3:**
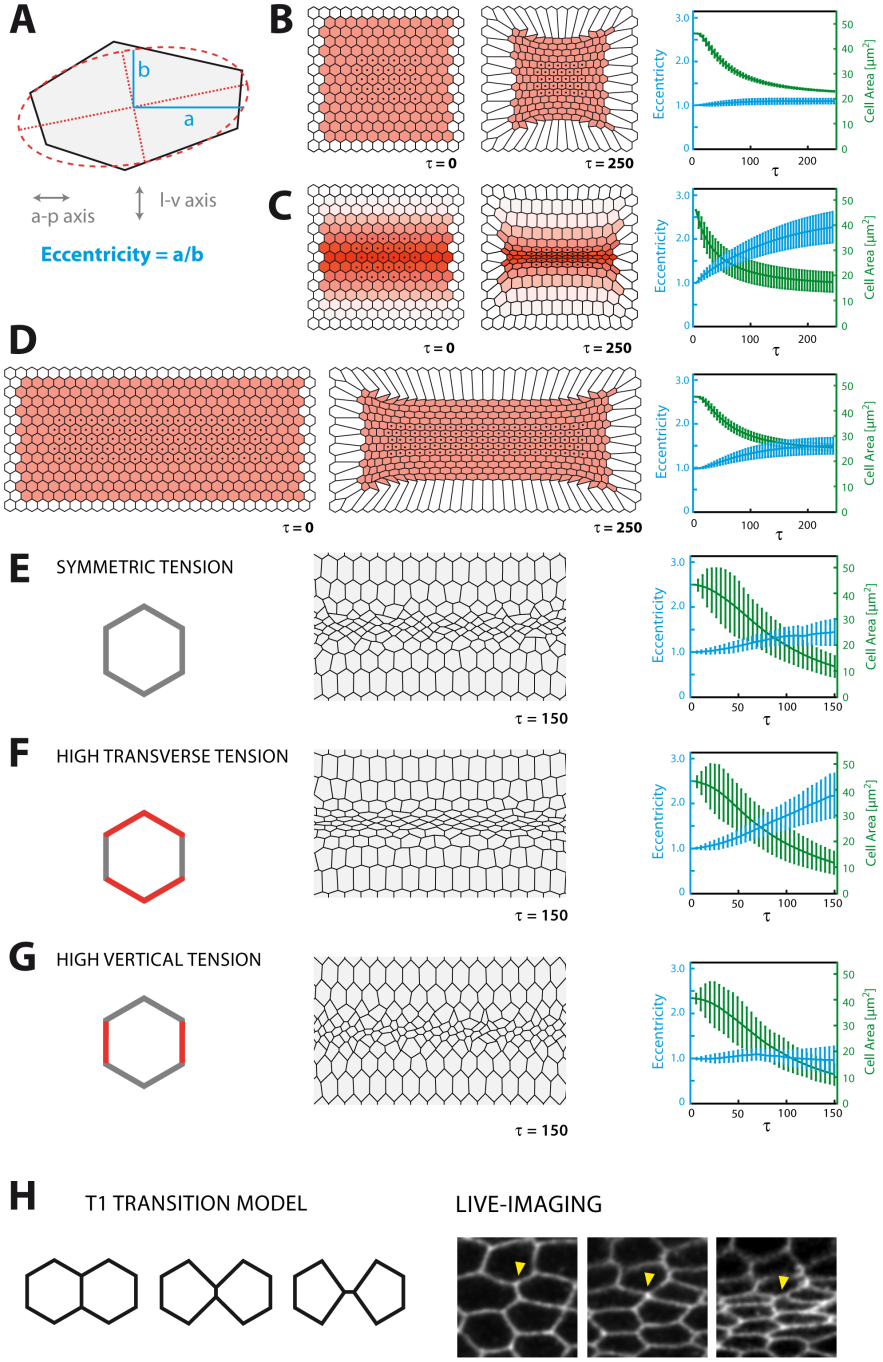
Anisotropic constriction. **A:** Anisotropy of constriction results in eccentric cell morphology that is quantified by relating the antero-posterior to the latero-ventral cell dimension after fitting an ellipse to the cell (after [21]). **B:** Model with constant contractility (red) on a square shaped sheet (white border cells non-contractile). **C:** Model with a gradient of contractility on the same cell sheet as in B. **D:** Model with constant contractility on a rectangular sheet (white border cells non-contractile). In B–D bar plots represent mean ± s.d. from the cells marked by black dots. **E:** Stochastic gradient model with standard parameters but symmetric line tension in all cells. Many cells undergo T1 transitions (see text and H) and remain uneccentric. **F:** Stochastic gradient model with standard parameters (transverse line tension assumed higher than vertical line tension). Cells grow markedly eccentric and T1 transitions remain rare. **G:** Stochastic gradient model with standard parameters but with vertical line tension assumed higher than transverse line tension. Like with symmetric tension, only minimal eccentricity is acquired. **H:** Model of T1 transitions (left). As two vertices approach each other, they swap neighbours. T1 transitions can be seen in live recordings of ventral furrow formation (right), but do not occur in high frequency.

With temporarily constant contractility enabled, both the cutoff and the gradient model result in the emergence of a band of constricted cells with lateral cells moving towards the midline, as seen in live-imaging data (Fig. 2A–C; Movies S1–S3). The degree of constriction as well as the morphological appearance of ventral cells are well reproduced but can change dramatically if alternative energy parameters are used (Figs. 3E–G, S1A–C). In any case, the cutoff model leads to a very artificial furrow morphology as strongly constricted cells of the central rows directly adjoin strongly dilated cells, located more lateral (Fig. 2A). Such an appearance is not seen in live-recordings where a more gradual transition from constricted to unconstricted cells can be observed (Fig. 2C; [22]). In contrast, the gradient model well reproduces this gradual shift and results in a more realistic overall morphology (Fig. 2B).

Next, we sought to let contractility vary in time. Live-imaging shows that apical myosin intensity starts weak in ventral cells and grows stronger during ventral furrow formation [6], [21](Fig. 2D). Thus, temporarily constant contractility appears not to be an appropriate assumption. We modelled increasing myosin activity by letting contractility start at zero at the onset of gastrulation and rise linearly in time (see Materials & Methods). Indeed, this adjustment adds a noticeable improvement to the model. Constriction now starts more subtle resembling *in vivo* data for which a slow rate of constriction at the onset of furrow formation has been documented (Movie S4) [22], [31]. However, all cells still follow the same deterministic time-course which is not seen in live-imaging where constriction appears more heterogeneous (Movie S1). Also, actomyosin contractions have been shown to occur in a very dynamical, asynchronous fashion [6] (Fig. 2D), which would not be well represented by a simple linear rise in contractility, identical in all cells. We introduced asynchronous contraction dynamics to our model by adding a Brownian motion to the linear term in order to include stochastic fluctuations in contractility (“stochastic gradient model”, see Materials & Methods). This way, each cell in the sheet executes individual contraction which follows a linear trend but features stochastic noise mimicking the dynamical action of the actomyosin (Fig. 2E; Movies S5, S6). Simulation of the stochastic gradient model yields very close resemblance to live-imaging data since a band of constricted cells is well reproduced and the more heterogeneous cell morphology realistically accounts for the appearance in live-recordings (Fig. 2D, 2E; Movie S1).

We conclude that a sharp border between contractile and non-contractile cells in the *Drosophila* ventral epithelium proves unlikely as such an arrangement would not match experimental data observed. In contrast, simulation of a contractility gradient, especially when combined with stochastic, asynchronous dynamics reproduces live-imaging data well and appears to be a good basis to computationally describe ventral furrow formation.

### The Model Predicts Anisotropic Constriction

While undergoing apical constriction, cells of the ventral furrow exhibit another conspicuous shape change as they gain an eccentric morphology [21], [31]. Apical constriction does not occur uniformly but asymmetrically (“anisotropically”, [21]) as cells reduce their latero-ventral diameter much stronger than the antero-posterior diameter (Figs. 2D,3A). As mentioned above, experimental evidence [21] as well as biomechanical analysis [30] point to asymmetrical tensions in the epithelium. Indeed, if tension is assumed to be identical in both the antero-posterior and latero-ventral axis, the simulation shows that cells will only yield marginal eccentricity while undergoing constriction as they will tend to undergo so-called T1 transitions [23], [33] and maintain a round shape, instead (Fig. 3E, 3H). In contrast, the simulation readily predicts eccentricity, i.e. anisotropic constriction, in ventral cells if transverse tension is assumed higher than vertical tension (Figs. 2E, 3F). However, additional features seem to be involved in the generation of anisotropic constriction since, despite higher transverse tension, we find constriction to occur without significant anisotropy when cells are arranged on a square-shaped sheet and contract with the same rate (Fig. 3B). Thus, we hypothesized that the geometry of the cell sheet as well as the spatial distribution of contractility may be additional parameters contributing to anisotropic constriction. For instance, when a contractility gradient is imposed on the square-shaped sheet, cells around the midline acquire distinct eccentricity (Fig. 3C). As contractility only varies along the latero-ventral axis in the gradient, contractile action along the antero-posterior axis will not differ between cells lying in the same row and will, consequently, balance out on average so little net constriction is achieved in this axis. Conversely, even under constant contractility, eccentricity can be achieved when the square-shaped cell sheet is expanded in the antero-posterior axis, mimicking the rectangular geometry of the *Drosophila* ventral epithelium (Fig. 3D). However, since it has remained unclear what causes tension to be asymmetrical in the ventral epithelium, it cannot be ruled out that the rectangular geometry may in fact imply higher tension along the antero-posterior axis, thus it may not be an independent cause of anisotropic constriction.

In any case, these results show that the model proves capable of correctly predicting anisotropic constriction and suggest that a contractility gradient adds another potential contribution to the generation of eccentric cell morphology during ventral furrow formation.

### The Model Predicts Incremental Cell Area Reduction

**Figure 4:**
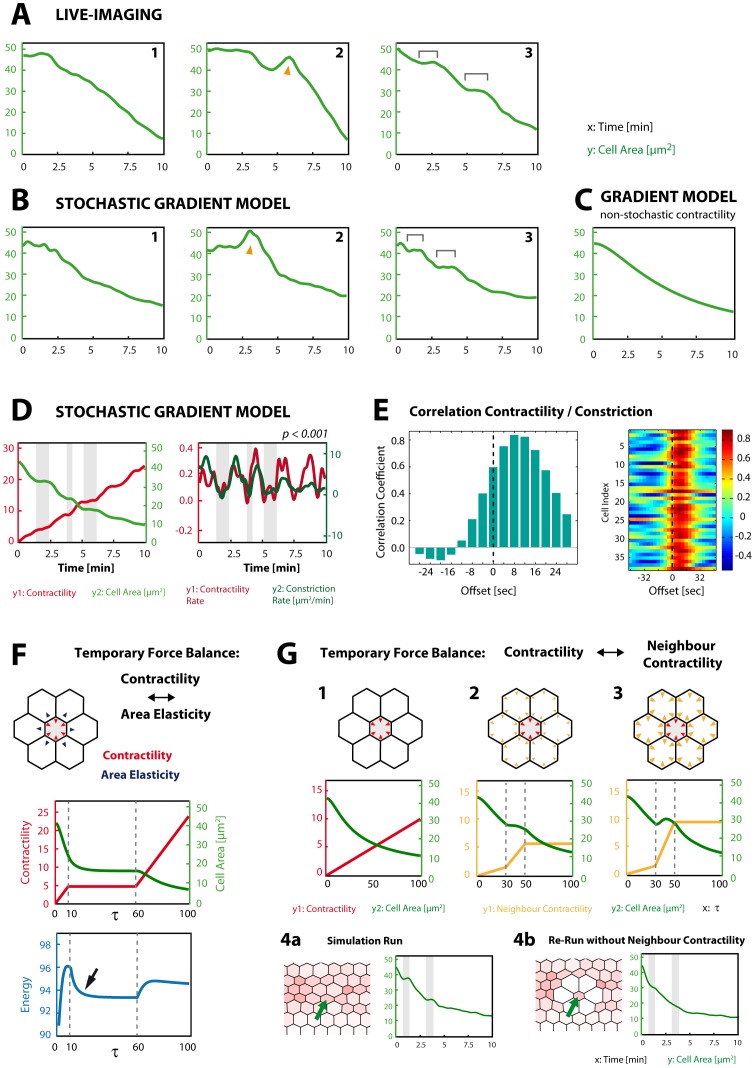
Analysis of incremental area reduction. **A:** Graphs of apical area typically obtained in live-recordings. Cells may become temporarily dilated (2, arrowhead) or show stagnation periods (3, brackets). Most graphs do not show unambiguous stagnations or other noticeably irregularities (1). **B:** Graphs of apical area typically obtained in the stochastic gradient model. Like in live-imaging, cells may show temporary dilations (2, arrowhead) and stagnation periods (3, brackets). **C:** Without stochastic fluctuations in contractility (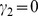, see Materials & Methods), all graphs of ventral cells equal each other, and stagnation periods are missing. **D:** Stagnation periods often coincide with periods of temporarily constant contractility (grey underlay) indicating correlation between contractility rate and area reduction rate ( = constriction rate). *p* represents significance of correlation. **E:** Correlation coefficients between contractility rate and constriction rate in a single cells (left) or for a total of 38 ventral cells (right), plotted against the offset by which contractility rate is shifted forward in time. Contractility rate precedes constriction by ca. 8 sec. (highest correlation), comparable to results obtained from live-imaging analysis (see Fig. 2e in [6]). Time designations in the model are obtained after normalizing the computational time-steps required to reach furrow completion to 10 min real-time (see Materials & Methods). **F:** Reduced model, consisting of one contractile central cell and six surrounding non-contractile cells. When contractility ceases to increase (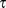 = 10), cell area reduction quickly stagnates as area elasticity balances contractility and total energy is locally minimized (arrow). Area reduction recommences only after contractility has begun to increase again (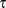 = 60) and exceeds elasticity. **G:** Reduced model like in F. When contractility of the central cell increases steadily with neighbour cells being non-contractile, area of the central cell is reduced without stagnations (1). When contractility of neighbour cells increases between 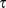 = 30 and 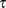 = 50, area reduction of the central cell stagnates (2). Depending on the strength of neighbour contractility increase, the cell can even become temporarily dilated (3). In (2) and (3) contractility of the central cell increases as in (1). (4a): Detail of a simulation run of the stochastic gradient model. Area and contractility of the marked cell (arrow) plotted to the right (grey underlays marking stagnation periods). (4b): When contractility of all surrounding cells is manually turned off, but otherwise the simulation is identically replicated, stagnations periods disappear or become vague.

We next investigated in more detail if the stochastic gradient model would quantitatively reproduce apical constriction. The stochastic contractility dynamics implemented in our model renders apical constriction highly variable, consistent with live-imaging analysis where the temporal dynamics of apical constriction also shows a high variability (Fig. 4A, 4B). The rate of constriction can vary considerably over time in a single cell, and cells may even show temporary dilations (Fig. 4B-2, arrowhead) – a phenomenon also seen in live-imaging analysis (Fig. 4A-2 arrowhead). A feature that has caught much attention in the analysis of ventral furrow formation *in vivo* is the appearance of stagnation periods – a time interval over which cell area remains nearly constant before area reduction recommences ([6]; Fig. 4A-3, brackets). It had been hypothesized that these stagnation periods represent a stereotypical phenomenon of apical constriction and are brought about by a cytoskeletal stabilization mechanism (ratchet) that is active between discontinuous contraction pulses [6]. This stabilization is supposed to be genetically controlled through Twi via an unknown mode of action [6]. Interestingly, stagnation periods readily show up in area graphs in the model (Fig. 4B-3, brackets). The construction of the model does not include a ratchet-like stabilization mechanism, thus we asked how the occurrence of distinct stagnations could be explained within the framework of our model. Stagnation periods are not seen if contractility rises linearly without stochastic fluctuation (Fig. 4C). Consequently, it is stringent to hypothesize that stochasticity in contractility may cause stagnations. Indeed, contractility rate and constriction rate show a significant correlation (Fig. 4D) strongly suggesting that stagnation in area reduction is linked to stagnation in contractility. It is noteworthy that contractility rate and constriction rate appear slightly shifted against each other (Fig. 4D): The correlation takes on its maximum if the contractility rate is slightly shifted forward in time (Fig. 4E). This phenomenon is in congruence with the cell responding to increasing intrinsic contractility by reducing its area and has also been found in live-imaging analysis [6]. We tested if temporary periods of stagnating contractility would imply stagnations in area reduction by considering a reduced model where we focused on a single cell surrounded by six neighbours (Fig. 4F). All cells in the reduced system have the same constants for area elasticity, line tension and line elasticity that were used for the standard simulations. However, contractility is only handed to the central cell and allowed to rise from zero in a step-like manner by implementing a period of constancy (Fig. 4F, 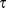 = 10 to 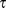 = 60). When equipped with this contractility dynamics, the central cell runs into a local “plateau” of constant area after contractility has ceased to rise and will recommence constriction only after contractility has begun to increase again (Fig. 4F, 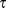 = 60). This observation becomes comprehensible when considering the energy in the system which will quickly fall to a local minimum after the cessation of contractility increase (Fig. 4F, arrow). Thus, stagnations are the consequence of temporary force balance between temporarily constant contractility and the opposing elastic forces within the cell. Due to the stochastic nature of contractility in the model temporary periods of nearly constant contractility may arise from time to time and will allow local force balance, resulting in stagnations in area reduction (Fig. 4D, grey underlays).

Although the intrinsic contractility can be well assumed to occur in a cell-autonomous fashion, constriction (or more general, cell shape change) will certainly not occur independently from adjacent cells since the cells are part of a mechanically coherent epithelium. Therefore, one has to take the possibility into account that contractions of adjacent cells may have a considerable effect on each other’s shape change. For instance, temporary dilations (i.e. increase of cell area), seen both in live-imaging analysis and in the model (Fig. 4A, 4B, arrowhead), are a likely consequence of the contractile activity of neighbouring cells due to their mechanical coherence. We sought to test the influence of neighbour contractility by first considering the reduced model in which we now allow neighbour cells to be contractile as well. In a reference simulation with the central cell featuring linearly rising contractility and no neighbour contractility, the area is reduced without stagnation (Fig. 4G-1). However, when we allow contractility in neighbour cells to rise, the contractility of the central cell will be counteracted to an extent which depends on the strength of neighbour contractility. Under moderate neighbour contractility the area of the central cell will stagnate (Fig. 4G-2), while under strong neighbour contractility it may even become temporarily dilated (Fig. 4G-3). This demonstrates that cell shape change cannot be considered independent from neighbouring cells in the epithelium. Indeed, neighbour contractility proves to have significant impact on cell shape of single cells when turning back from the reduced model to the stochastic gradient model: Simulation runs of the model were recorded in which a cell showed stagnation periods and therefore incremental area reduction. When these simulation runs were repeated under identical settings, except that the contractile activity of the neighbouring cells were manually set to zero, stagnation periods and incremental area reduction were lost (Fig. 4G-4a,b).

Based on these finding we assume that analogous mechanisms work during apical constriction in ventral furrow formation *in vivo*. As cells are tightly coupled via adherens junctions and execute asynchronous stochastic actomyosin contractions, cell constriction will be constrained by contraction activity of neighbouring cells. Consequently, we hypothesize that stagnations in area reduction could be explained through the counterbalancing effects of elastic forces within the cell and the contractile action of neighbouring cells.

### *twi* Mutants can be Modelled with Randomly Reduced Contractility

**Figure 5:**
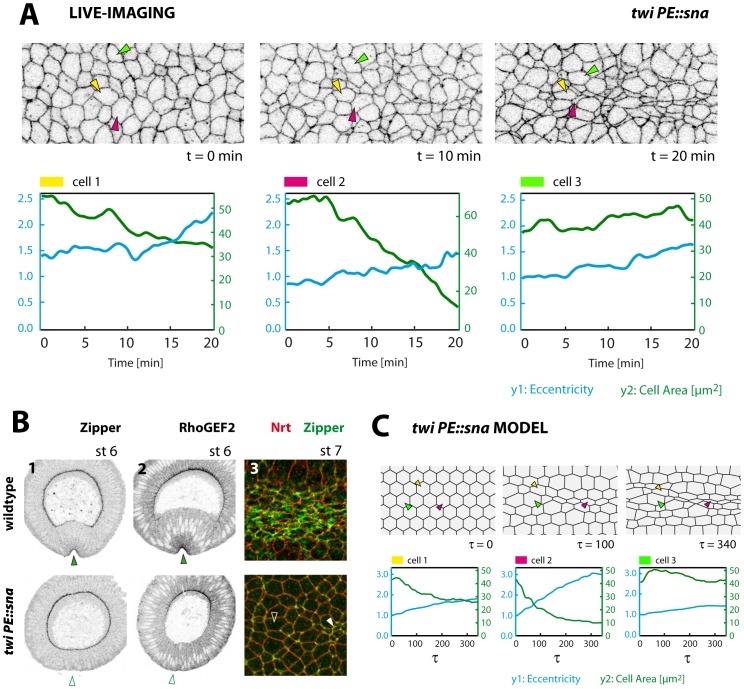
Ventral furrow phenotype of *twi* mutants. **A:** Stills of a live-recording of a *twi* mutant spanning 20 minutes after completion of cellularization (Spider:GFP). Plots depict eccentricity and apical area for three indicated cells. **B:** Localization of myosin (Zip) and RhoGEF2 in wildtype and *twi* mutant embryos, fixed during ventral furrow formation. 1 and 2 cross-sections (stage 6), 3 surface section (2 µm below surface, stage 7). Myosin and RhoGEF2 localize to the apices in ventral cells in the wildtype (1,2: arrowheads). In *twi* this apical localization fails at stage 6 (1,2: empty arrowheads). At stage 7 low levels of myosin accumulate apically, but only in a subset of ventral cells (3: filled arrowhead, compare to cell under empty arrowhead) **C:** Stills of a simulation run with reduced contractility, varying randomly in the sheet (see Materials & Methods). The simulation resembles the experimental observations in *twi* mutants with a random arrangement of incompletely constricted (yellow), strongly constricted (red) or unconstricted cells (green) (compare to A).

**Figure 6:**
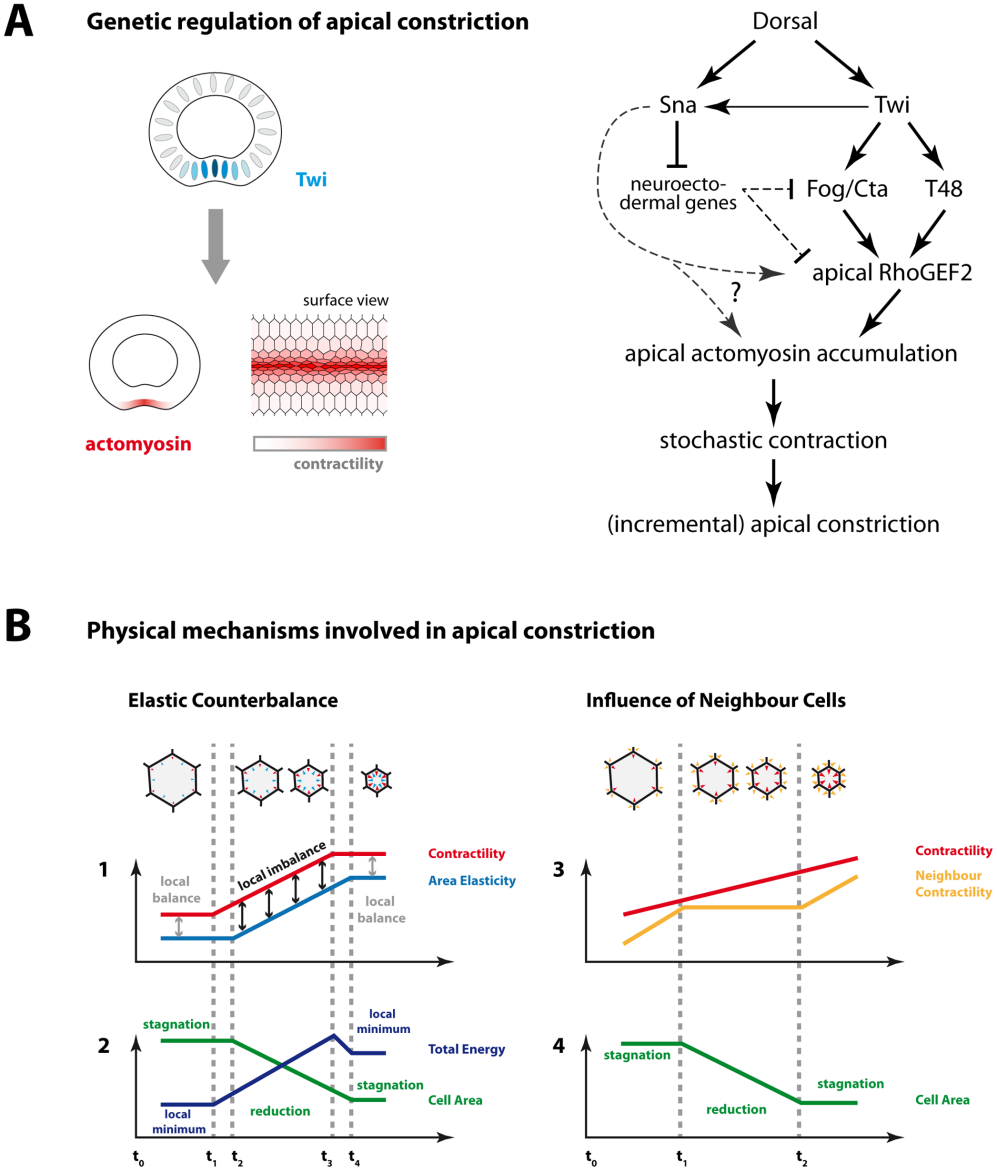
Proposed mechanisms driving apical constriction during ventral furrow formation. **A:** Genetic cascade (modified after [36]) leading from the dorso-ventral determinant Dorsal to shape change of ventral cells (right). Twi activates downstream targets (Fog, T48) leading to the apical accumulation of contractile actomyosin. Stochastic actomyosin contractions then reduce apical cell area. Area reduction may exhibit an incremental fashion due to stochastic interplay with opposing forces (see B). We suppose that the graded expression of Twi translates into graded accumulation of apical actomyosin and thus graded contractility (left). Twi also enhances Sna expression, but the nature of the Sna*-*dependent contribution to apical actomyosin assembly (seen in *twi PE::sna* mutants, Fig. 5B) remains to be resolved. It is open whether there is some positive input via an unknown target of Sna or whether inhibition of neuroectodermal gene expression by Sna releases a repression of apical actomyosin accumulation. **B:** Two physical mechanisms contributing to incremental area reduction, as suggested by the computational model: Intrinsic elastic counterbalance (left) and extrinsic contractive counterforce exerted by neighbour cells (right). Both mechanisms may work alternatively or in concert to cause temporary stagnations in area reduction. Left: As long as contractile and elastic forces are balanced, cell area remains constant (1,2: t_0_ to t_1_). When contractility begins to rise (1: t_1_), the cell responds by reducing its area after a short delay (2: t_2_). As a consequence of this area reduction, i.e. compression, elastic energy begins to rise (1: t_2_). When contractility has ceased to rise (1: t_3_), the cell arrives at a new area level where contractile and elastic forces are in balance and total energy is locally minimized (1,2: t_4_). Right: Contractive forces exerted by neighbouring cells can temporarily outweigh the contractive force in the central cell preventing net area reduction (1,2: t_0_ to t_1_ and t_2_ onwards). In the graph, “neighbour contraction” refers to the average contractive forces taken over all adjacent cells.

Finally, we wanted to test if our gradient model would also prove sufficient to reproduce mutant phenotypes. Twi acts a major regulator of ventral furrow formation as upon loss of Twi the ventral furrow is severely compromised and does not invaginate [34]. Indeed, apical constriction is largely missing in *twi* mutants with only a subset of ventral cells undergoing area reduction which is also considerably slowed down (Fig. 5A). However, unlike previously stated [6] we did not find cell area to markedly oscillate between reduction and dilation (Fig. 5A; Movie S7). In particular, temporary dilations are rare and are also observed in wildtype (see above, Fig. 4A). Immunostaining reveals that myosin fails to properly localize to the apices in ventral cells upon the onset of gastrulation at stage 6 in *twi* mutants but remains stuck at the basal sides (Fig. 5B-1). Also RhoGEF2, being a critical requirement for apical actomyosin localization, does not properly localize to the apices in *twi* mutants ([5], see also Fig. 5B-2) – as expected based on the genetic model of furrow formation (Fig. 6A). Faint apical localization of myosin is visible not earlier than stage 7, but occurs fragmentarily as only a subset of cells accumulate detectable myosin (Fig. 5B-3). Thus, we assume that apical constriction in *twi* mutants is largely absent because of incomplete actomyosin localization – in concordance with the presumed role of Twi as a master regulator of ventral furrow formation [34](Fig. 6A). We sought to model this weak, fragmented myosin accumulation by randomly scaling down contractility within the cell sheet while letting all other parameters unaltered (see Materials & Methods). Thus, contractility is now generally lowered, but this defect affects different cells to a different degree. Introducing this variability turns out to be sufficient to disrupt the formation of the ventral furrow in the model. Ventral cells now constrict to a variable extent leading to a highly irregular appearance, as seen in live recordings (Fig. 5C; Movie S8). Both constricted and unconstricted cells can be found in a random spatial arrangement. As in live-imaging, eccentricity is markedly reduced among cells (Fig. 5C), possibly because many cells can now undergo unconstrained constriction due to the reduced counterforce exerted by neighbour cells which fail to contract. Thus, the model shows that randomly reduced contractility as derived from experimental data proves sufficient to explain the irregular constriction in *twi* mutants and the disruption of the ventral furrow.

## Discussion

Computational modelling serves as a valuable addition to the methods toolbox when investigating a complex developmental process like ventral furrow formation. In this study, we adopt a well-established vertex model to computationally describe apical constriction during ventral furrow formation. We find that the ventral furrow is realistically reproduced in the model if contractility is assumed to follow a latero-ventral gradient in the ventral epithelium and cells execute stochastic contraction dynamics. The model predicts that constriction will be anisotropic, demonstrating that cells are forced into eccentric morphology due to the physical constraints in the epithelium and do not require intrinsic polarization. Moreover, the model predicts that constriction may temporarily stagnate during the course of furrow formation leading to incremental cell area reduction. Analysis of the model reveals that this incremental area reduction occurs passively as a result of opposing forces arising from elasticity and from contraction of adjacent cells. In particular, it is not required to postulate an active stabilization mechanism to achieve incremental area reduction in the model. Analysis of *twi* mutants does not make a stabilization mechanism a mandatory postulate *in vivo*, either. Therefore, we conclude that the model presented here serves as a promising basis for a parsimonious concept of apical constriction during ventral furrow formation.

### A Contractility Gradient may Drive Ventral Furrow Formation

The state-of-the-art genetic model of ventral furrow formation [1], [2], [4], [5], [35], [36] states that the maternal ventral fate determinant Dorsal directs ventrally confined expression of the two zygotic ventral fate determinants Twi and Sna which function as master activators of ventral furrow formation (Fig. 6A). Twi acts through two downstream pathways (Fog/Cta and T48) to achieve the apical accumulation of RhoGEF2 which in turn triggers apical accumulation of contractile actomyosin [2], [3], [5]. Measurements of myosin intensity in fixed or live specimen unequivocally show that actomyosin activity is not equal throughout the ventral epithelium but is higher the closer the cell is located to the ventral midline (Fig. 1F–H). In fact, the apparent heterogeneity of constriction levels prior to furrow invagination [22], [34]; Fig. 2C; Movie S1), i.e. cells close to the midline being noticeably more constricted than those lying further lateral, makes the notion of a contractility gradient a very reasonable concept. Considering the epistasis of ventral furrow formation (Fig. 6A), it is plausible to hypothesize that the graded expression of Twi [32] might translate into the graded activation of Fog [4], and indirectly of RhoGEF2 and finally myosin (Figs. 1F–H, 6A). In this scenario the contractility of a cell would depend on the expression level of Twi, thus the cell’s latero-ventral position during cellularization would determine its contractility during furrow formation in a cell-autonomous fashion. Although we favour this view as a parsimonious hypothesis concerning the experimental data and the current genetic model of furrow formation, we are aware that is has not been directly shown what factors control the quantitative amount of contractility in ventral cells. One might, therefore, contemplate an alternative gradient model by which contractility is determined as a function of distance to the midline, e.g. controlled through a, yet to be identified, secreted molecule. Fog would be a plausible candidate since its apical secretion might implicate a non-cell-autonomous mode of contractility induction. In this scenario, cells would grow more contractile the closer they approach the midline during furrow formation (Fig. S1E), instead of being predetermined through their Twi expression level. It is noteworthy that this alternative gradient model leads to comparable results since simulations also achieve the formation of a furrow which, however, tends to be wider than observed in our gradient model (Fig. S1E). As cells approach the midline, differences in contractility will decrease so, eventually, a broader range of cells will have nearly identical high contractility. In this sense, this alternative gradient model would, in fact, be an intermediate between our gradient model and the cutoff model. Importantly, the phenomena addressed in this study, such as eccentricity and incremental area reduction, occur likewise in this alternative gradient model and, thus, do not depend on the molecular determination of cell contractility (Fig. S1E). Another alternative hypothesis to explain actomyosin contraction being present in ventral cells but absent in lateral cells, would be a sharp border separating contractile from non-contractile cells – possibly mediated by the cutoff-like expression pattern of Sna [32]. As shown in our model, this hypothesis leads to an artificial morphology and does not match live data (Fig. 2A).

We therefore propose that contractility in the ventral epithelium follows a gradual pattern *in vivo*, as predicted by the computational model and supported by myosin intensity measurements. As we do not have a method at hand to quantitatively relate contractility to myosin signal (pixel intensity), the graded contractility in our model is a qualitative concept whose numerical parameters must be chosen to match the experimental data. Lower overall contractility, for instance, will only yield low constriction until force balance is reached, so only an inconspicuous furrow is formed (Fig. S1B, also see Fig. 1C). Similarly, narrowing or widening the contractility gradient implies very narrow or very wide furrows which are typically not seen in live recordings (Fig. S1C, S1D; compare Fig. 2C). As for the alternative gradient model (see above), occurrence and causes of incremental area reduction do not depend on either overall contractility or gradient width (Fig. S1A–D). A previous computational approach [19] also briefly mentioned the hypothesis of a gradient of contraction probability together with the hypothesis of graded nuclear expression of Sna. However, the molecular role of Sna in ventral furrow formation remains still enigmatic since its known function of repressing neuroectodermal genes gives no explanation why Sna is absolutely critical for apical actomyosin localization and shape change in ventral cells [6], [34], [36](Fig. 6A).

### Genetic Definition of the Spatiotemporal Domain of the Ventral Furrow

Twi has been proposed to serve an additional, albeit uncharacterized, function during ventral furrow formation by controlling a cytoskeletal ratchet necessary to stabilize constricted cell apices during area reduction [6]. However, it appears questionable whether a specific loss of such a postulated stabilization mechanism is unambiguously evident based on the *twi* phenotype since oscillations of apical area in constricting cells seem hardly more excessive in *twi* mutants compared to wildtype (Fig. 5A; Movie S7). Presumably, a qualitative difference between amorphic *twi* mutants and hypomorphic *twi*-RNAi contributes to the discrepancy to the data shown in [6]. Interestingly, the *twi* phenotype can be mimicked in our computational model if contractility is reduced (Fig. 5C) – a modelling concept being in congruence with experimental data since apical actomyosin localization is severely affected as predicted by the genetic model (Figs. 5B,6A). We are aware, though, that our model does not yet fully reproduce live recordings of *twi* mutants because cell constrictions appear to occur in a wider spatial domain whereas they are restricted to around five cell rows in the wildtype (compare Movies S1 and S7). This can be reproduced in the simulation by widening the contractility gradient (Movie S9), however the genetic model cannot explain such a widening of the contractile domain upon loss of *twi* yet. A similar phenomenon can be recognized in germline clones of *rap1*, where cell constrictions are also only seen in a subset of ventral cells but occur in a wider domain in the ventral epithelium (see Fig. 2 and Movie S2 in [8]). It remains to be clarified what causes this widening of the contractile domain and whether this is due to genetic or physical implications. It also needs to be resolved why cells still accomplish remaining, albeit delayed and fragmentary, apical myosin localization in the absence of Twi (Fig. 5B-3). This myosin localization occurs via an unexplored mechanism, but must depend on Sna (Fig. 6A) since no apical myosin is seen in *sna twi* double mutants at any time (not shown). Possibly, Sna equips all cells within its expression domain (which is wider than the ventral furrow) with a minute basic contractility (Fig. 6A, dashed pointers). A Twi-dependent strong, graded contractility may superimpose this basic contractility and may determine the spatial range of constriction under regular circumstances.

Consequently, we favour to adhere to the genetic model in Fig. 6A as it sufficiently accounts for results gained from both experimental and modelling approaches so far. In particular, we prefer not to postulate an additional role of Twi in stabilization of contracting actomyosin since we feel that the phenotype does not make this conclusion sufficiently coercive. In fact, our vertex model demonstrates that incremental area reduction occurs in a frequency comparable to *in* *vivo* and without rigorous direct regulation of alternating contraction and stabilization periods. Thus, in order to minimize complexity and to keep the genetic model parsimonious, it should first be considered whether occasional stagnation periods seen in live-imaging analysis could be merely physical phenomena as suggested by the vertex model (Fig. 6B) rather than manifestations of a genetically controlled mechanism.

### Conclusion and Outlook

We suggest that the joint constriction of a band of cells in the epithelium seen during ventral furrow formation is best regarded as the outcome of stochastic autonomous contractions which are genetically constrained to a short time-slot and to a limited spatial domain. A genetic cascade facilitates a gradual apical accumulation of contractile actomyosin which contracts in a stochastic fashion to reduce apical cell area. These contractions are carried out autonomously in each cell and are opposed by elastic resistance within the cell as well as by contractions of neighbouring cells. As our model shows, no further regulatory input like a ratchet mechanism is required to achieve joint cell constriction in the epithelium with occasional stagnation of area reduction in individual cells. Laser ablation experiments could possibly help to quantify the extent by which adjacent cells affect each other’s shape change *in vivo*. Similar experiments had shown that mechanical coupling between contracting cells in the amnioserosa largely affect shape changes of adjacent cells during dorsal closure [37].

It has not been fully understood what mechanical role the joint apical constriction plays for tissue invagination. Absent or severely disturbed apical constriction as seen in *sna*, *twi, RhoGEF2* or *rap1* mutants prevents tissue invagination [8], [34], [35]. However, computational approaches have suggested that apical constriction alone is not sufficient to achieve furrow invagination since the lateral epithelium may in fact be a critical driving force to guarantee regular tissue internalization [10], [12]. Joint constriction may cause a small indentation in the ventral epithelium which will subsequently act as a weak spot or predetermined breaking line allowing quick tissue invagination by the sudden release of pressure in the epithelium. Extension of the vertex model into the third dimension will be a promising endeavour to investigate this process and will further elucidate the mechanisms by which tissues undergo a massive morphogenetic movement like ventral furrow formation.

## Materials and Methods

### Vertex Modelling of the Ventral Furrow

In order to model cell shape change during ventral furrow formation we set-up a variant of a commonly used vertex model (compare e.g.: [23], [26], [27], [28]) to describe epithelia during development. The corresponding energy function is explained in Fig. 1. Parameter values were chosen by fitting the model to the cell morphology seen in live-recordings. Elasticity parameter K and preferred area A_0_ were assumed constant and identical for all cells (K = 2.5). Line tension 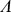 was supposed to be asymmetrical with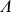 = 0.2 for transverse boundaries (angle between 0° and 45° in relation to antero-posterior axis) and 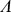 = 0.075 for vertical boundaries (angle between 45° and 90° in relation to antero-posterior axis). Standard size of the cell sheet is 15×24 cells (cropped only for illustration purpose).

For the cutoff model, contractility (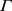) was modelled by setting 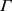 = 10 in five central cell rows and 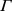 = 0 on the remaining sheet. For the gradient model contractility was modelled using the function 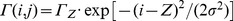 with 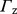 being contractility in the midline ( = central cell row *Z*) (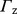 *_= _*15), *i* the cell row (latero-ventral coordinate) and *j* the cell column (antero-posterior coordinate) and 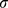 the width parameter of the gradient (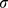 = 2.0). To introduce time-dependence in the cutoff model, we used 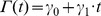 in the five central cell rows and 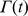 = 0 elsewhere (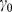 = 0, 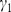
_ = _0.15). Time-dependence in the gradient model was achieved by using 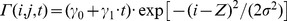. The linear term was bounded by 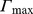
_ = _25 to avoid unlimited increase of contractility. Finally, for the stochastic gradient model autonomous stochastic dynamics were implemented by adding Brownian motion to the contractility function via
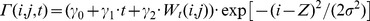
where 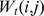 represents a path of a standard Wiener process, drawn independently for each cell (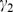 = 0.3). To model incomplete apical myosin accumulation in *twi PE::sna* mutants, contractility in the formula above was modified via 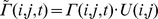 with *U(i,j)* being a uniform random number between 0.0 and 0.5, drawn independently for each cell.

Each vertex in the model moves following the directive to minimize the energy in the sheet. To derive the equation of motion for each vertex, we assume balance between frictional force and potential force as described previously in [25]. The equations are numerically integrated using an explicit Euler scheme. The simulations can either be run for a fixed number of time-steps or continue until the width of the furrow ( = the average area of cells from the five central most rows) falls below a certain threshold. Boundary vertices remain fixed through time. At the left and right margins of the sheet two cell columns (only one in Fig. 3B–D) are set non-contractile (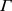 = 0 throughout) to delimit the anterior and posterior borders of the furrow. To reduce border artefacts, line tension (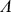) is set to 6.0 in all cells of the non-contractile margin. If the distance between two vertices falls below a critical threshold *eps* (*eps* = 0.1), they undergo a swapping process (“T1 transition”) by changing connections with adjacent vertices (Fig. 3H, also see Fig. 2 in [25], Fig. 1b in [28] or Fig. S1 in [23]). Real-time designations in the model as displayed in Fig. 4 are obtained by normalizing the time-steps required to reach completion of furrow formation to real-time in live-recordings (10 minutes). Area designations in the model are obtained by normalizing the pixel area of the initial regular hexagon to the average area of cells at the end of cellularization *in vivo*. All data from a simulation run (including the stochastic contractility dynamics) are saved to disk so simulation runs can be identically replicated or replicated after directed manual modifications like in Fig. 4–4b. The model is implemented as a MATLAB script (The MathWorks).

### Fly Stocks & Antibodies

*Spider:GFP* [38]; *sqh:mCherry* [6] (*spaghetti-squash (sqh)* encoding myosin regulatory light chain); *twist^EY53^* [39]; *P[sna_g_]* [40] (*PE::sna,* this construct drives *sna* expression using a *twi*-independent enhancer element, so a *twi* loss-of-function will not be superimposed by additional partial loss of *sna)*. rabbit anti-RhoGEF2 (1∶10000, [41]), rabbit anti-Zipper (1∶1000, [42], *zipper* encoding non-muscle myosin heavy chain), mouse anti-Nrt (BP106; 1∶10, DSHB), GAM-Cy5 (1∶500, Jackson Labs), GAR-Cy3 (1∶500, Jackson Labs).

### Microscopy and Image Analysis

For antibody staining and cross-section imaging, embryos were heat-fixed using standard procedures and manually cut with a needle in AquaPolymount (Polysciences). For live-imaging, dechorionated embryos were glued ventral side up onto a slide and covered in water-saturated 3 S Voltalef oil. Image series were recorded on a Leica SP2 IRBE at 8 or 12 sec./frame starting from the onset of gastrulation (cellularization front has reached the yolk). Scanning depth was 2 µm below the apical surface. Automated image analysis (segmentation and cell tracking) was performed like in [8], using custom MATLAB scripts (The MathWorks). Intensity measurements with manual ROIs were done in IPLab (Scanalytics) or ImageJ (NIH).

## Supporting Information

Figure S1
**Variations of the gradient model. A:** Stochastic gradient model with standard parameters. **B:** Stochastic gradient model with reduced overall contractility (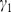 = 0.075, 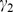 = 0.15, 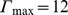). Only an inconspicuous furrow is achieved. **C,D:** Stochastic gradient model with a narrow or a wide gradient (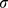 = 1.25 or 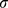 = 3.00, resp.). The furrow ends up slimmer or wider than typically seen in live-recordings. Independent from overall contractility or gradient width, cells can be found which execute incremental area reduction (A–D, brackets). **E:** Alternative gradient model. This model equals the stochastic gradient model, except that the contractility of a cell is not calculated on the basis of its row index *i* via 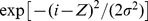 but via 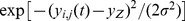 instead, with 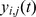 being the *y*-coordinate of the centroid of the cell at time-step *t* and 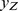 being the ventral midline ( = mean *y*-coordinate of the centroid of the central cell row at time-step 1). This way, the cell’s contractility will increase the further it approaches the midline. The model shows similar performance as the stochastic gradient model with cells gaining eccentricity and undergoing constriction with or without stagnation periods, but with a slightly wider furrow.(TIF)

Movie S1 **Ventral Furrow Formation.** Confocal live-recording of the ventral epithelium of a *Drosophila* embryo expressing Spider:GFP to mark cell membranes. Imaging plane 2 µm below the apical surface. Movie accelerated 120×, spanning 10 minutes real-time.
(MOV)

Movie S2
**Cutoff Model.** Model of ventral furrow formation using a cutoff contractility function and standard parameters (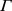 = 10 in contractile cells, 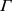 = 0 in non-contractile cells, contractility constant in time).(MOV)

Movie S3
**Gradient Model.** Model of ventral furrow formation using a gradient contractility function and standard parameters (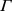 = 15 in the ventral midline, contractility constant in time).(MOV)

Movie S4
**Time-dependent Gradient Model.** Model of ventral furrow formation using a time-dependent gradient contractility function (without stochastic fluctuation) and standard parameters (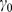 = 0, 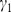 = 0.15, 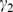 = 0.0, see Materials & Methods).(MOV)

Movie S5
**Stochastic Gradient Model.** Model of ventral furrow formation using a time-dependent gradient contractility function with stochastic fluctuations (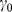 = 0, 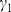 = 0.15, 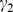 = 0.3, see Materials & Methods). For short, this model is referred to as “Stochastic gradient model” throughout the article.(MOV)

Movie S6 **Stochastic Gradient Model, with contractility coded by colour.** Same model as in Movie S5. Stochastic cell contractility is coded by colour, ranging from white (zero contractility) to dark red (maximum contractility).
(MOV)

Movie S7 **Phenotype of** ***twi*** **mutants.** Confocal live-recording of the ventral epithelium of a *twi* mutant embryo (*twi PE::sna*) expressing Spider:GFP to mark cell membranes. Imaging plane 2 µm below the apical surface. Movie accelerated 120×, spanning 20 minutes real-time.
(MOV)

Movie S8 ***twi*** **Model.** Model of ventral furrow formation in *twi* mutants using random reduction of contractility across the cell sheet and standard parameters otherwise (see Materials & Methods).
(MOV)

Movie S9
**Modified** ***twi*** **Model.** Model of ventral furrow formation in *twi* mutants using random reduction of contractility across the cell sheet and a widened contractility gradient (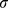 = 4.25, standard parameters otherwise).(MOV)
