# Supplementary figures and images for: A Vertex Model of Drosophila Ventral Furrow Formation
Source: PLoS One. 2013 Sep 16;8(9):e75051. doi: 10.1371/journal.pone.0075051 (PMC3774731; doi:10.1371/journal.pone.0075051)

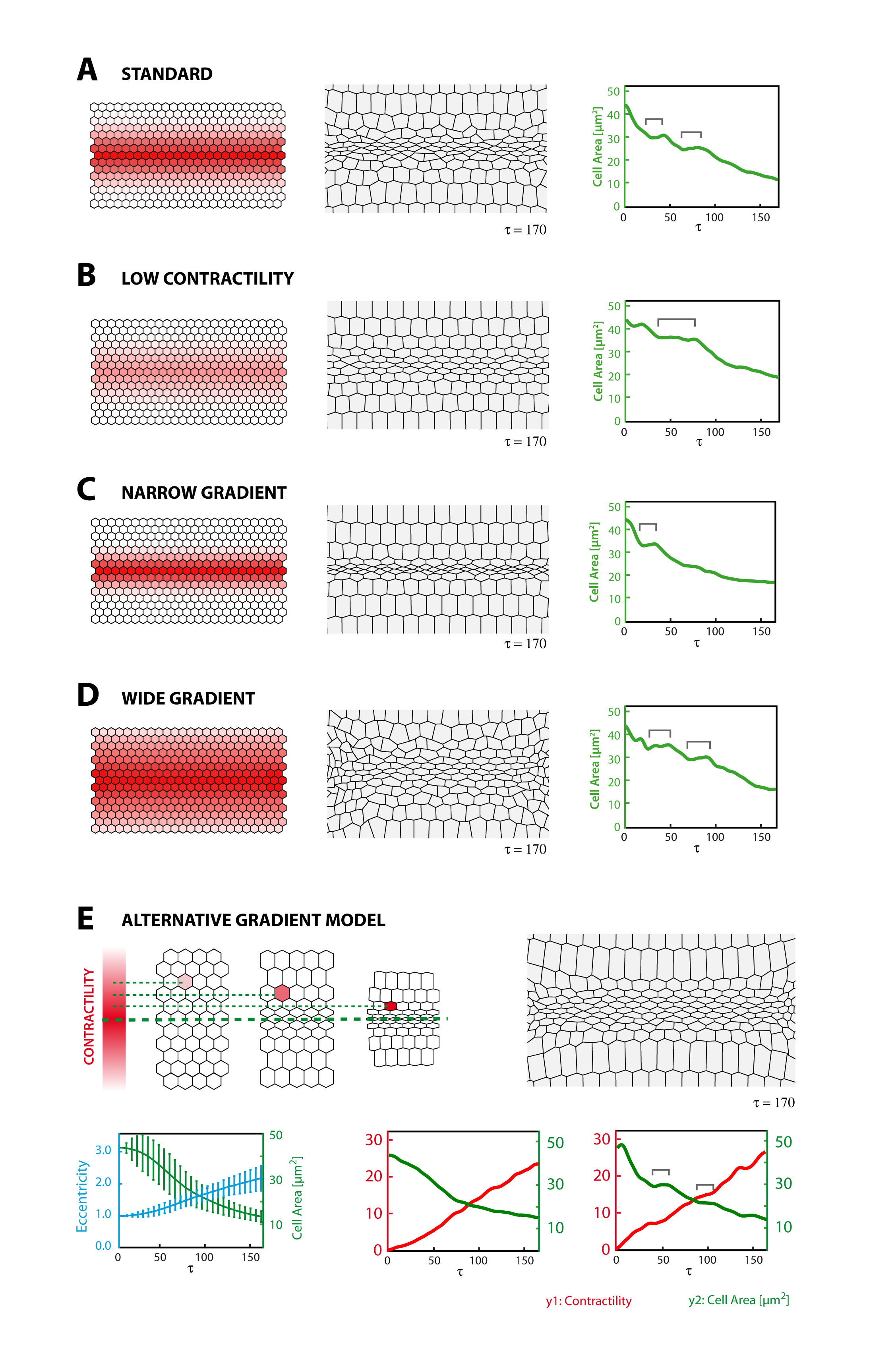

Supplement: Figure S1 — Variations of the gradient model. A: Stochastic gradient model with standard parameters. B: Stochastic gradient model with reduced overall contractility ( = 0.075, = 0.15, ). Only an inconspicuous furrow is achieved. C,D: Stochastic gradient model with a narrow or a wide gradient ( = 1.25 or = 3.00, resp.). The furrow ends up slimmer or wider than typically seen in live-recordings. Independent from overall contractility or gradient width, cells can be found which execute incremental area reduction (A–D, brackets). E: Alternative gradient model. This model equals the stochastic gradient model, except that the contractility of a cell is not calculated on the basis of its row index i via but via instead, with being the y-coordinate of the centroid of the cell at time-step t and being the ventral midline ( = mean y-coordinate of the centroid of the central cell row at time-step 1). This way, the cell’s contractility will increase the further it approaches the midline. The model shows similar performance as the stochastic gradient model with cells gaining eccentricity and undergoing constriction with or without stagnation periods, but with a slightly wider furrow. (TIF) [file pone.0075051.s001.tif]
